# Behind the Wall—Compartment-Specific Neovascularisation during Post-Stroke Recovery in Mice

**DOI:** 10.3390/cells11101659

**Published:** 2022-05-17

**Authors:** Anja Kolbinger, Roxane Isabelle Kestner, Lara Jencio, Tim J. Schäufele, Rajkumar Vutukuri, Waltraud Pfeilschifter, Klaus Scholich

**Affiliations:** 1 Institute of Clinical Pharmacology, *pharmazentrum frankfurt* Goethe-University, D-60590 Frankfurt am Main, Germany; kolbinger@med.uni-frankfurt.de (A.K.); schaeufele@med.uni-frankfurt.de (T.J.S.); 2 Department of Neurology, Hospital of the Goethe University Frankfurt, Theodor-Stern-Kai 7, D-60590 Frankfurt am Main, Germany; roxane-isabelle.kestner@kgu.de (R.I.K.); lara.jencio@gmx.de (L.J.); 3 Institute of Pharmacology and Toxicology, *pharmazentrum frankfurt* Goethe-University, D-60590 Frankfurt am Main, Germany; vutukuri@med.uni-frankfurt.de (R.V.); waltraud.pfeilschifter@klinikum-lueneburg.de (W.P.); 4 Department of Neurology and Clinical Neurophysiology, Municipal Hospital Lüneburg, D-21339 Lüneburg, Germany; 5 Fraunhofer Institute for Translational Medicine and Pharmacology ITMP, Theodor-Stern-Kai 7, D-60596 Frankfurt am Main, Germany; 6 Fraunhofer Cluster of Excellence for Immune-Mediated Diseases CIMD, Theodor-Stern-Kai 7, D-60596 Frankfurt am Main, Germany

**Keywords:** stroke, angiogenesis, dendritic cell, microglia, T-cell, multiplex immunohistochemistry

## Abstract

Ischemic stroke is a highly prevalent vascular disease leading to oxygen- and glucose deprivation in the brain. In response, ischemia-induced neovascularization occurs, which is supported by circulating CD34^+^ endothelial progenitor cells. Here, we used the transient middle cerebral artery occlusion (tMCAO) mouse model to characterize the spatio-temporal alterations within the ischemic core from the acute to the chronic phase using multiple-epitope-ligand cartography (MELC) for sequential immunohistochemistry. We found that around 14 days post-stroke, significant angiogenesis occurs in the ischemic core, as determined by the presence of CD31^+^/CD34^+^ double-positive endothelial cells. This neovascularization was accompanied by the recruitment of CD4^+^ T-cells and dendritic cells as well as IBA1^+^ and IBA1^−^ microglia. Neighborhood analysis identified, besides pericytes only for T-cells and dendritic cells, a statistically significant distribution as direct neighbors of CD31^+^/CD34^+^ endothelial cells, suggesting a role for these cells in aiding angiogenesis. This process was distinct from neovascularization of the peri-infarct area as it was separated by a broad astroglial scar. At day 28 post-stroke, the scar had emerged towards the cortical periphery, which seems to give rise to a neuronal regeneration within the peri-infarct area. Meanwhile, the ischemic core has condensed to a highly vascularized subpial region adjacent to the leptomeningeal compartment. In conclusion, in the course of chronic post-stroke regeneration, the astroglial scar serves as a seal between two immunologically active compartments—the peri-infarct area and the ischemic core—which exhibit distinct processes of neovascularization as a central feature of post-stroke tissue remodeling. Based on our findings, we propose that neovascularization of the ischemic core comprises arteriogenesis as well as angiogenesis originating from the leptomenigeal vasculature.

## 1. Introduction

Ischemic stroke is a highly prevalent vascular disease that carries a relevant burden of disability and socioeconomic strain. It is a consequence of the acute occlusion of a cerebral artery leading to oxygen- and glucose deprivation in the brain parenchyma of the respective vascular territory. In this context, the activation of microglia and rapid deterioration of neurons occurs, leading to the release of damage-associated molecular pattern molecules (DAMPS) and thereby to further glial activation and subsequent secretion of cytokines and matrix metalloproteinases [1]. As a result, disruption of the blood–brain barrier (BBB) takes place, which paves the way for immune cell infiltration into the otherwise strictly immunologically privileged brain [2]. In the course of astrocyte activation, a broad glial scar forms around the ischemic border, virtually sealing the ischemic core (IC) off from the peri-infarct area. Whether it represents a protective mechanism keeping the surviving parenchyma separated from the inflamed and necrotic tissue or whether this separation in fact hinders regenerative processes to expand into the ischemic core is not yet clearly understood [3,4]. 

So far, studies on post-stroke recovery focused on the peri-infarct area because it is considered to represent the closest histological correlate of the penumbra. The penumbra was characterized in 1977 by Astrup et al. as a region where blood flow has dropped below the threshold necessary to uphold electrophysiological neuronal functions, but not yet below the threshold necessary to maintain structural integrity, retaining the potential of full recovery in case of reperfusion within a certain time window [5]. Today, all causal stroke therapies rely on this concept.

Furthermore, significant regenerative potential is attributed to the peri-infarct area, which is reflected, e.g., by the occurrence of strong ischemia-induced neovascularization [6]. This term summarizes three possible mechanisms: (a) *vasculogenesis*, the sprouting of new vasculature mainly taking place during embryogenesis and tumor formation, but also observed to a certain extent in cardio-vascular diseases [7]; (b) *arteriogenesis*, which means the enlargement of pre-existing collaterals mainly driven by shear stress sensed by the affected endothelium [8]; and (c) *angiogenesis*, where a stress signal such as ischemia activates endothelial cells to form new vessels, which is supported by circulating endothelial progenitor cells (EPCs) [9]. These EPCs share several characteristics with hematopoietic stem cells, e.g., the expression of hematopoietic progenitor cell antigen CD34 [10]. They stimulate or potentially even integrate into the activated endothelium of pre-existing vessels to differentiate into tip cells, which clear and guide the way for new sprouts through the surrounding extracellular matrix [11]. Tip cells are followed by stalk cells, which show high proliferative activity to elongate the forming vessel and create a vascular lumen and branches [12]. Supported by macrophages, tip cells subsequently fuse with other tip cells to build up vascular circuits [13]. During maturation, the newly formed vessels attract and integrate pericytes and other mural cells [14]. 

Although these newly formed vessels during post-stroke recovery have shown to lack a fully equipped BBB and possibly recede over the long term [15], angiogenesis has been associated with functional recovery [16]. It has been proposed that angiogenesis could support neural remodeling by stimulating neural progenitor cell proliferation through secreted mediators such as VEGF or BDNF [17] serving as scaffolds for the migration of those cells from the subventricular zone (SVZ) and supplier of nutrients and oxygen to enhance neural maturation [18]. Whether these processes also take place within the ischemic core remains elusive. So far, this compartment has received less attention as it is usually considered the necrotic remnant of the ischemic event. 

Nevertheless, evidence is accumulating that certain aspects of the post-stroke inflammatory process are specific to this region [19,20]. It has been argued that stroke patients’ outcomes rely less on the extent of the penumbra, which is relatively uniform between cases, and more on the varying volume of the ischemic core [21]. Multiple attempts to design immunomodulatory treatments for ischemic stroke have not yet translated into clinical therapeutic strategies. Therefore, a compartment-specific approach might be key to future research targets. 

We therefore aimed at a comprehensive characterization of the spatio-temporal alterations within the ischemic core from the acute to the chronic phase after experimental stroke using a multiple-epitope immunohistochemical approach. For this purpose, mice were nursed following an intensive post-stroke care protocol [22] and examined clinically and histologically at 1, 3, 7, 14 and 28 days after transient middle cerebral artery occlusion (tMCAO). 

## 2. Materials and Methods

### 2.1. Ethics Statement

All animals involved in the presented experiments were approved by the local Ethics Committees for Animal Research (Darmstadt) under the permit number FU/1182. Experiments were performed in accordance with the German Protection of Animals Act and the guidelines for care and use of laboratory animals by the local committee (Regierungspräsidium Darmstadt).

### 2.2. Transient Middle Cerebral Artery Occlusion and Post-Stroke Care

**Figure 1:**
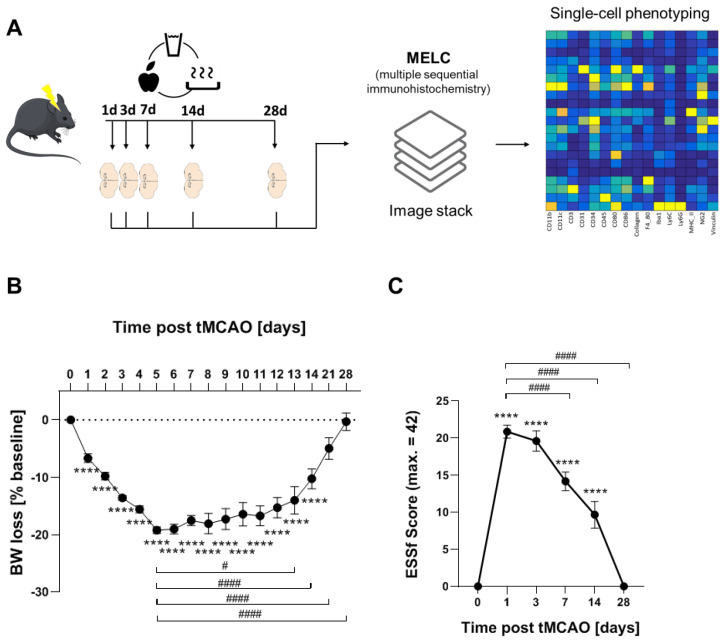
Long-term recovery in the tMCAO model. (**A**) Flow chart of study design. (**B**) Weight loss tMCAO surgery presented as percentage reduction from preoperative body weight ± SEM n = 25, **** *p* < 0.0001 vs. baseline, # *p* < 0.05, #### *p* < 0.0001 vs. d5. One-way ANOVA with Bartlett correction. (**C**) ESSf scores for mice taken at the indicated days after stroke induction ± SEM. n = 25, **** *p* < 0.0001 vs. baseline, #### *p* < 0.0001 vs. d5. One-way ANOVA with Bartlett correction.

In total, 25 male C57Bl/6J mice (25–30 g) at the age of 10 to 12 weeks old were purchased from Charles River Laboratories (Sulzfeld, Germany). The right middle cerebral artery was occluded as described previously [23]. In brief, mice were anesthetized using 1.5% isoflurane administered continuously via a breathing mask and kept on a temperature-controlled heating mat throughout surgeries. The right common and external carotid arteries were ligated before the insertion of a standard silicone-coated monofilament (6-0 medium, coating length 9 mm, catalog No. 6023910PK10; Doccol, Sharon, MA, USA) until the branching point of the middle cerebral artery. After 1 h, the filament was retracted to allow reperfusion. To achieve analgesia 0.1 mg/kg bodyweight buprenorphine was injected i.p. 30 min before surgery and every 8 h during the first 48 h. Up to 5 animals were grouped into cages and kept on a heating mat (Beurer, Ulm, Germany). The body weight, rectal temperature and clinical signs of pain, agitation or severe apathy were monitored daily. Functional scoring was performed on day 1, 3, 7, 14 and 28 after surgery, using the Experimental Stroke Scale (ESSf) developed by Lourbopoulos et al. [22]. This score comprises 11 items testing for sensory and motor dysfunction, neglect, behavioral and coordination deficits. To secure an adequate food and water supply, we followed the maximized post-stroke support protocol described before including easily accessible jelly food, oral feeding support and s.c. injections of warmed 0.9% saline adjusted to the animals’ individual demand. The mortality rate was 24% including 3 mice that were excluded due to signs of herniation, subarachnoid hemorrhage, or late-onset ischemia. Surviving animals were followed-up for 24 h, 3 d, 7 d, 14 d or 28 d post-stroke, grouping 5 animals per observation timepoint. All procedures have been conducted in an unblinded and unrandomized manner as all animals received the same treatment and no intervention (e.g., drug administration) was performed. A total of 4 animals per group were used for MELC analysis. An overview of the study design is given in Figure 1A. All brains were examined macroscopically for signs of subarachnoid hemorrhage after removal. Tissue sections were macroscopically examined for blood-clot formations.

### 2.3. Multi-Epitope-Ligand-Carthographie (MELC)

MELC technology is an automated immunohistological imaging method and can be used to visualize very high numbers of antibodies on the same sample as described before [24,25,26]. Briefly, whole brains were isolated and natively frozen in Tissue TEK O.C.T. Compound (Sakura Finetek, Torrance, CA, USA) on dry ice and stored at −80 °C until sectioning. Mid-brain coronal tissue sections were taken at a 10 µm thickness on silanized cover slips, fixed in 4% paraformaldehyde in PBS for 15 min, permeabilized with 0.1% Triton X100 in PBS for 15 min and blocked with 3% BSA in PBS for 1 h. The sample was placed on the stage of a Leica DM IRE2 and a picture was taken. Then, in an automated procedure, the sample was incubated for 15 min with bleachable fluorescence-labelled antibodies and rinsed with PBS. Afterward, the phase contrast and fluorescence signals were imaged by a cooled charge-coupled device camera (Apogee KX4, Apogee Instruments, Logan, UT, USA). A bleaching step was performed to delete fluorescence signals, and the post-bleaching image was recorded. Then, the next antibody was applied and the process was repeated. For data analysis, fluorescence images produced by each antibody were aligned pixel-wise and were corrected for illumination faults using flat-field correction. The post-bleaching images were subtracted from their following fluorescence image. The antibodies used are listed in Supplemental Table S1.

### 2.4. Analysis of MELC Data

In the first step, all greyscale antibody channel images were processed using ImageJ 1.52v to diminish noise, background fluorescence and remove artefacts for further analyses if necessary. The staining for NeuN, GFAP and Map2 were used to divide the images in areas containing stroke or healthy tissue. Subsequently, Cell Profiler (version 3.1.9) [27] was used for additional illumination correction and the generation of a cell mask for single-cell segmentation using the images for propidium iodide (cell nuclei) and CD45. The resulting segmentation mask was loaded into histoCAT (version 1.76) (20) together with the corresponding antibody channel images. All images, excluding the images used for single-cell mask generation, were z-score normalized and used for Barnes–Hut t-SNE (BH t-SNE) [28] and PhenoGraph analysis [29] as implemented in histoCAT. PhenoGraph defines cell clusters based on single-cell mask and marker colocalization (k was set to 20 or 30). A BH t-SNE scatter plot was overlaid with a colored PhenoGraph cluster map. The clusters of the PhenoGraph were exported from histoCAT and further analysed with the SPADE V3.0 tool for Matlab to generate spanning trees of density-normalized events [30]. After defining the single cells with the segmentation mask in histoCAT, the z-score normalized images were exported and used for a FACS-like analysis in FlowJo (version 10.8.1). To investigate the relationship between clusters, neighborhood analysis under standard conditions as implemented in histoCAT was used [31]. The gained result was a score between 0 and 100 for all clusters which was imported in Cytoscape (version 3.8.2) to generate neighborhood trees [32].

### 2.5. Data Analysis and Statistics

All data are presented as mean ± SEM. Determination of statistically significant difference in all experiments including was conducted with one-way analysis of variance (ANOVA) followed by post hoc Bonferroni correction using GraphPad Prism. 7. For in vitro experiments comparing only two groups, Student’s *t*-test was carried out with Welch’s correction. A *p* value of <0.05 was considered statistically significant.

## 3. Results

### 3.1. Long-Term Recovery from Stroke

Using a recently established protocol for murine post-stroke care, we were able to achieve full recovery in 76% of the mice in the tMCAO model [22,33]. The mice undergoing tMCAO showed a weight loss of up to 20% after stroke induction whereby the weight loss reached the nadir at day 5. From day 6 onward, weight either stabilized or started to recover (Figure 1A). tMCAO-induced focal neurological or systemic behavioral deficits were measured using the experimental stroke scale (ESSf) [22]. The ESSf score was maximal after 1 day post-stroke and receded completely until day 28 post-stroke (Figure 1B).

To investigate the time course of neovascularization and immune cell recruitment in the tMCAO model, we used the MELC technology for multiple sequential immunohistochemistry. The MELC technology is an automated system, allowing the imaging of an unrestricted number of directly labelled antibodies on the same tissue sample. Overall, 26 antibodies were used to image vessels, neurons, glia cells and immune cells (Supplementary Table S1). Since the cellular and immunological responses in the stroke area are expected to undergo changes between stroke induction and full recovery, we tested the expression of these markers at times, which define the acute (day 1, 3 post-stroke), subacute (day 7) and the recovery phase (day 14) as well as day 28 where full recovery was achieved according to bodyweight and ESSf scores.

**Figure 2:**
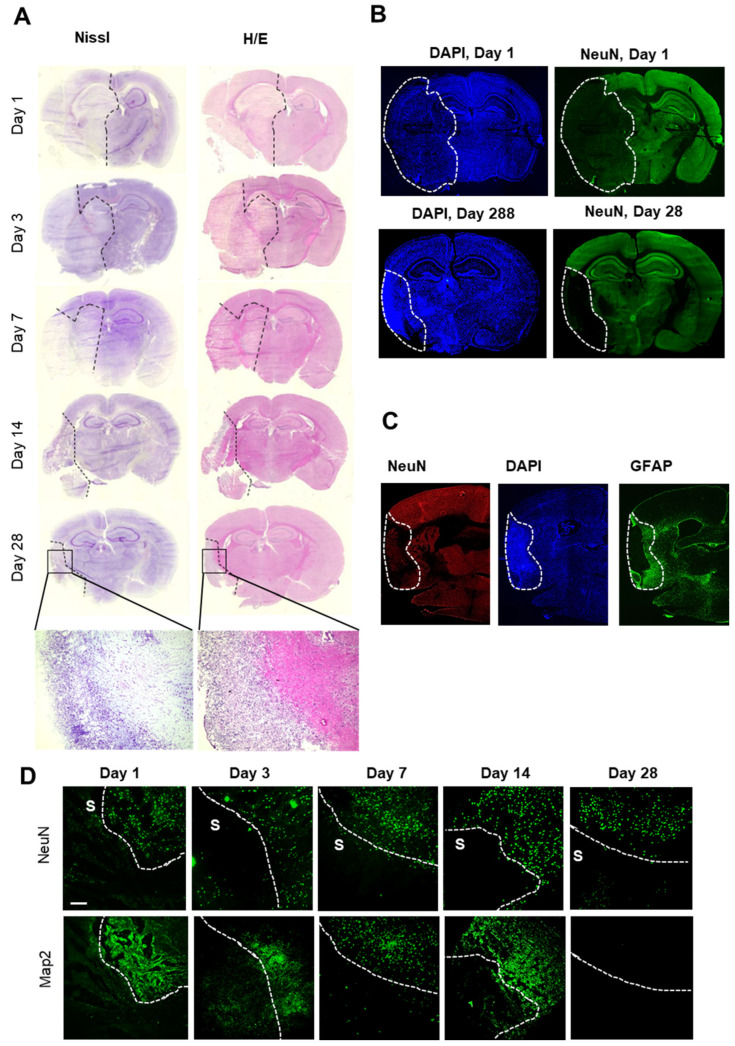
Histochemical and immunohistochemical analyses of the stroke area. (**A**) Nissl- and H/E-staining of brains at the indicated times after stroke induction. The black dashed lines depict regions with severe cell loss. (**B**) Immunohistochemical staining for nuclei (DAPI) and neurons (NeuN). The white lines depict areas with severe neuronal loss. (**C**) Immunohistochemical staining for nuclei (DAPI), astrocytes (GFAP) and neurons (NeuN). The white dotted lines depict the area with severe neuronal loss (ischemic core). (**D**) Immunohistochemical staining of areas at the border between stroke (S) and unaffected tissue for MAP2 and NeuN. The white dotted lines depict the border between stroke and unaffected tissue. The white bar represents 100 µm.

In a first step, brain slices were stained with either cresyl violet or hematoxylin/eosin stain to define stroke areas. Cresyl violet and hematoxylin/eosin staining showed a dramatic cellular loss in the right hemisphere starting 1 day after stroke; thereafter, the affected area decreased significantly over time towards a defined subpial cortical region (Figure 2A). Twenty-eight days after stroke induction, this region showed intense nuclear staining but only light cytoplasmic staining (Figure 2A). Surprisingly, a striking structural reorganization of the peri-infarct area including partial repopulation by neurons seemed to occur (Figure 2B). Immunofluorescent staining for GFAP confirmed the formation of a wide astroglial scar surrounding the ischemic core at the border to the peri-infarct area (Figure 2C), showing a strict compartmentalization of the ischemic hemisphere during long-term observation after tMCAO.

In accordance with previous reports, the ischemic area was defined by the absence of neurons and low expression of MAP2 (i.e., day 3–14 after stroke) [34,35] (Figure 2D). Thus, in the following experiments, the combined information from histological and immunohistochemical staining for neurons (NeuN) and the microtubule-associated protein 2 (MAP2) [34,35] was used to define the border of the stroke-affected area, which were chosen for the MELC analysis (Figure 2D).

### 3.2. Temporal and Spatial Patterns of Neoangiogenesis during Stroke Recovery

**Figure 3:**
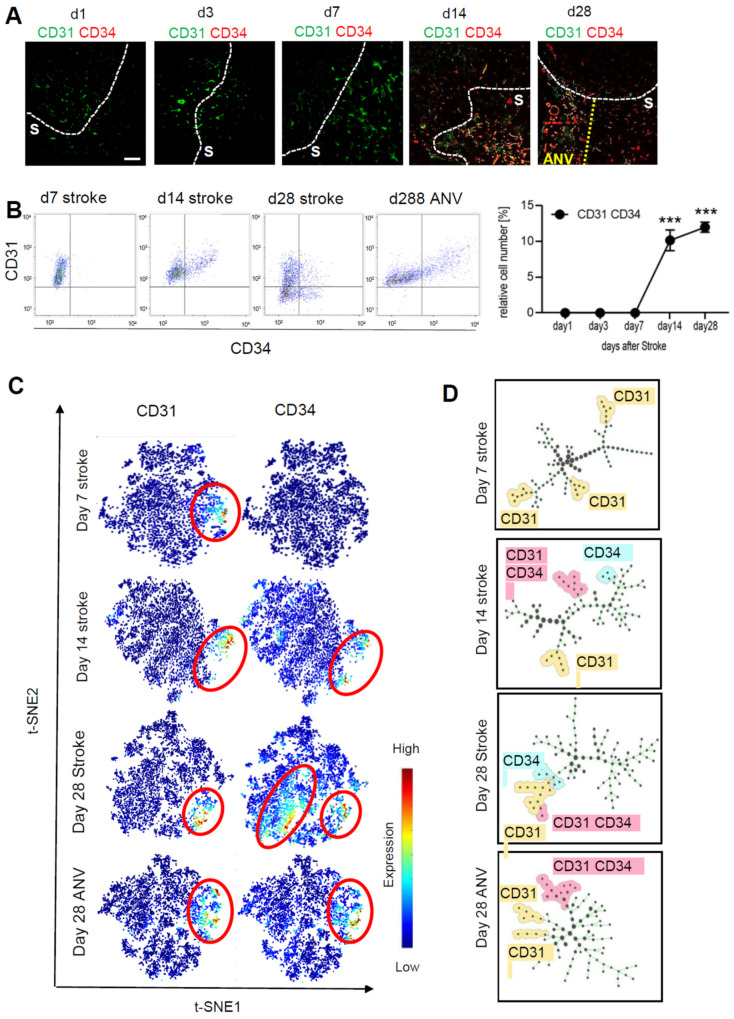
Analysis of neoangiogenesis over time of the stroke area. (**A**) Representative MELC images showing CD31 and CD34 staining in healthy tissue, stroke area (S) and the ischemic core area of neovascularization (IC-ANV) at the indicated times. The white bar represents 100 µm. (**B**) Quantitative assessment of CD31^+^/CD34^+^ endothelial cells based on the MELC images using flow cytometry standard format in the ischemic core and IC-ANV. Data are shown as mean ± S.E.M (n = 4). One-way ANOVA/Bonferroni *** *p* < 0.001. (**C**) tSNE maps with highlighted CD31 and CD34 expression at the indicated times. (**D**) Identification of cell subpopulations and cell transitions using SPADE analysis at the indicated times in the ischemic core and IC-ANV.

To determine neovascularization, we analyzed the expression of CD31^+^/CD34^+^ vessels over the course of 28 days in the stroke area using the MELC technology. The fields of vision for MELC analyses were chosen at the border of the stroke area to compare vascularization in the ischemic core and peri-infarct area. For quantitative assessment of CD31^+^/CD34^+^ endothelial tip cells, we used a segmentation mask to extract single-cell data from images including abundances of all measured markers and the microenvironment of each cell such as cell neighbors and cell crowding. This information was compiled into a flow cytometry standard format (.fcs) file for further analysis, which showed a strong upregulation of CD31^+^/CD34^+^ endothelial cells starting at day 14 after stroke induction (Figure 3A,B). Interestingly, at day 28, the granulomatous, subpially localized cortical remnants of the ischemic core exhibited an especially high amount of neovascularization and is in the following referred to as “ischemic core area of neovascularization” (IC-ANV; Figure 3A,B). Subsequent analysis using tSNE maps as a multi-dimensional reduction tool also showed a high amount of non-endothelial stem cells (CD31^−^/CD34^+^) in the ischemic core regions, apart from the CD31^+^/CD34^+^-populated IC-ANV (Figure 3C). The identification of cell subpopulations and cell transitions using spanning-tree progression analysis of density-normalized events (SPADE) confirmed the increased presence of the CD31^+^/CD34^+^ population in the IC-ANV as compared to the surrounding ischemic core (Figure 3D). Thus, so far, the data show that angiogenesis, as defined by the occurrence of CD31^+^/CD34^+^ endothelial tip cells, starts in the areas affected by the stroke 14 days after induction. Twenty-eight days post-ischemia, an especially strong neovascularisation is observed in the subpial cortical area, which might be due to a massive outgrowth of leptomeningeal vessels.

### 3.3. Temporal and Spatial Patterns of Immune Cell Recruitment

**Figure 4:**
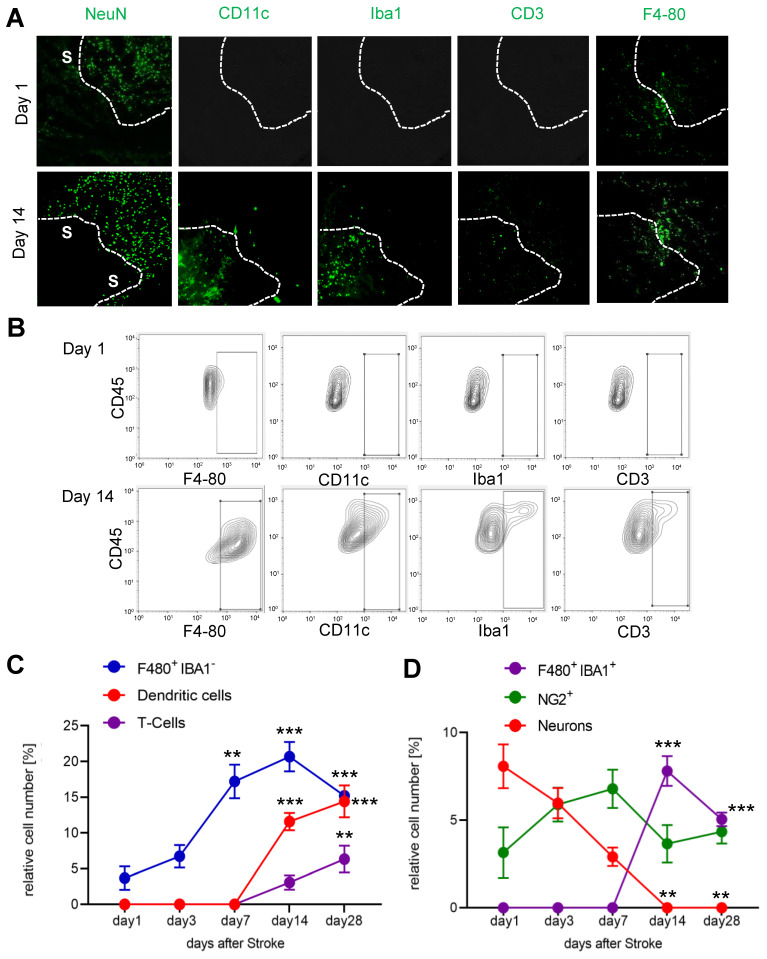
Analysis of immune cell recruitment to the stroke area. (**A**) Representative MELC images showing stroke-affected (S) and unaffected areas at day 1 and 14 after stroke induction. The white lines depict the borders between the two areas. The white bar represents 100 µm. (**B**–**D**) Quantitative analysis of immune cells based on the MELC images using flow cytometry standard format in the stroke-affected regions. Data are shown as mean ± S.E.M. (n = 4). One-way ANOVA/Bonferroni ** *p* < 0.01; *** *p* < 0.001.

Since we observed an increased cell density in the IC-ANV and it has been shown that several immune cell types, such as macrophages and neutrophils, can induce or support post-stroke angiogenesis [36], we investigated the recruitment of immune cells to the ischemic core and especially their presence in the IC-ANV. Therefore, we performed MELC runs using antibodies to identify endothelial cells, microglia, monocyte-derived macrophages, dendritic cells, neutrophils, and various T-cell subtypes (Supplemental Table S1). We found that until day 7 post-stroke, only microglia (F4-80^+^/Ly6C^−^/Iba1^−^) were detectable in the ischemic core (Figure 4A–C). However, starting at day 14 post-stroke, dendritic cells, T-cells and Iba1-expressing microglia also appeared in the stroke-affected region (Figure 4D). The number of neurons decreased rapidly after stroke induction (Figure 4B,D), while NG2-positve cells (probably oligodendrocyte precursor cells (OPCs) and pericytes) remained relatively stable over time (Figure 4B,D).

**Figure 5:**
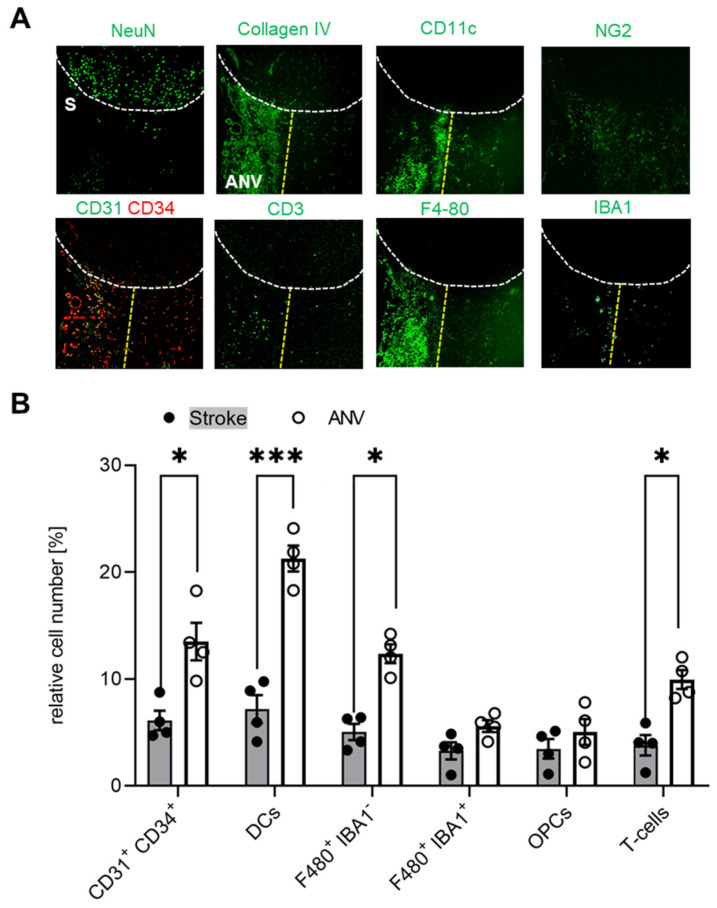
Localized differences of the immune cell distribution in the stroke-affected region. (**A**) Representative MELC images of different immune cells in unaffected and stroke-affected areas (S) as well as IC-ANV 28 days after stroke induction. White dotted lines depict the borders between the three areas. The white bar represents 100 µm. (**B**) Quantitative analysis of immune cells based on the MELC images using flow cytometry standard format in the ischemic core and IC-ANV. Data are shown as mean ± S.E.M (n = 4). Two tailed Student’s *t*-test * *p* < 0.05; *** *p* < 0.001.

Since we found that the subpial cortical IC-ANV showed a higher cell density as compared to the surrounding ischemic area (Figure 2B), in the next step, we investigated whether or not immune cell recruitment is increased in this area. Quantitative analysis of the MELC data confirmed that the number of CD31^+^/CD34^+^ endothelial tip cells was significantly increased in the IC-ANV as compared to neighboring stroke-affected areas, mainly comprising the astroglial scar (Figure 5A,B). In regard to the number of immune cells, we found that dendritic cells, Iba1-negative microglia, and T-cells showed a roughly twofold increase in the IC-ANV as compared to neighboring stroke-affected areas (Figure 5A,B). In contrast, the number of NG2^+^ cells and pericytes and Iba1-positive microglia did not differ between both regions (Figure 5A,B).

**Figure 6:**
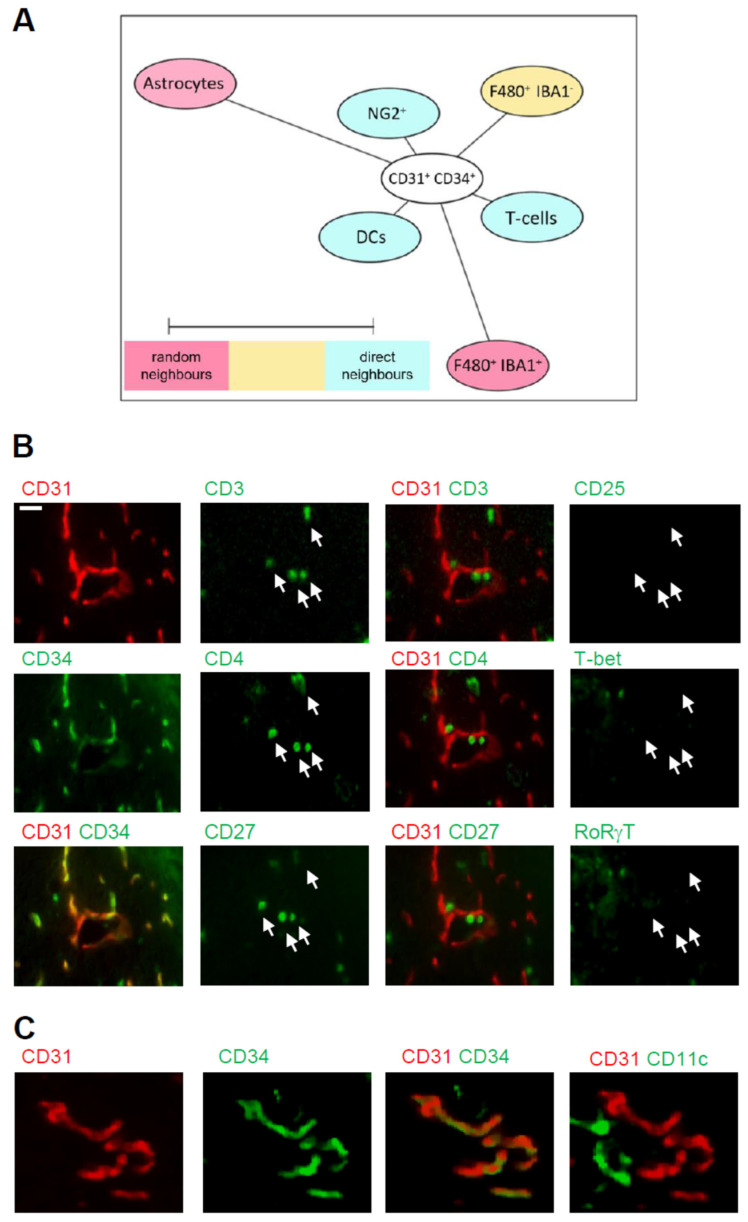
Cellular neighborhood of CD31^+^/CD34^+^ endothelial cells in the IC-ANV. (**A**) Network visualization using Cytoscape of the cellular neighborhood of CD31^+^/CD34^+^ endothelial cells in the IC-ANV 28 days after stroke induction. The distance between CD31^+^/CD34^+^ cells and the other cell types in the visualization represents the statistical probability of being direct neighbors. (**B**,**C**) Representative MELC images showing T-cells (panel (**B**)) and dendritic cells (panel (**C**)) in the neighborhood of CD31^+^/CD34^+^ endothelial cells in the IC-ANV at the indicated times. The white bar represents 10 µm.

To study which of these cell types are putative interaction partners of CD31^+^/CD34^+^ endothelial tip cells and could influence angiogenesis, we aimed to determine the cellular neighborhood of these cells. Therefore, we performed a neighborhood analysis [31], which aims to identify cell types neighboring the CD31^+^/CD34^+^ endothelial cells more often, as expected for a random distribution. The scores received by the neighborhood analysis were then plotted using Cytoscape software to visualize the data as neighborhood trees (Figure 6A). The neighborhood analysis showed that dendritic cells, T-cells, and NG2-positive cells, which most likely represent pericytes, were direct neighbors of CD31^+^/CD34^+^ endothelial tip cells, while microglia and astrocytes were more rarely in the neighborhood of the endothelial tip cells (Figure 6A). The T-cell population consisted of CD3^+^/CD4^+^/CD27^+^/CD8^−^/T-bet^−^/RORγt^−^ expressing T-cells (Figure 6B) and the dendritic cells CD11c^+^/F4 80^−^ expressing cells (Figure 6C). Taken together, the data show an accumulation of immune cells in the IC-ANV including dendritic cells and CD4 T-cells, which neighbor the CD31^+^/CD34^+^ endothelial tip cells and therefore might support post-stroke angiogenesis.

## 4. Discussion

**Figure 7:**
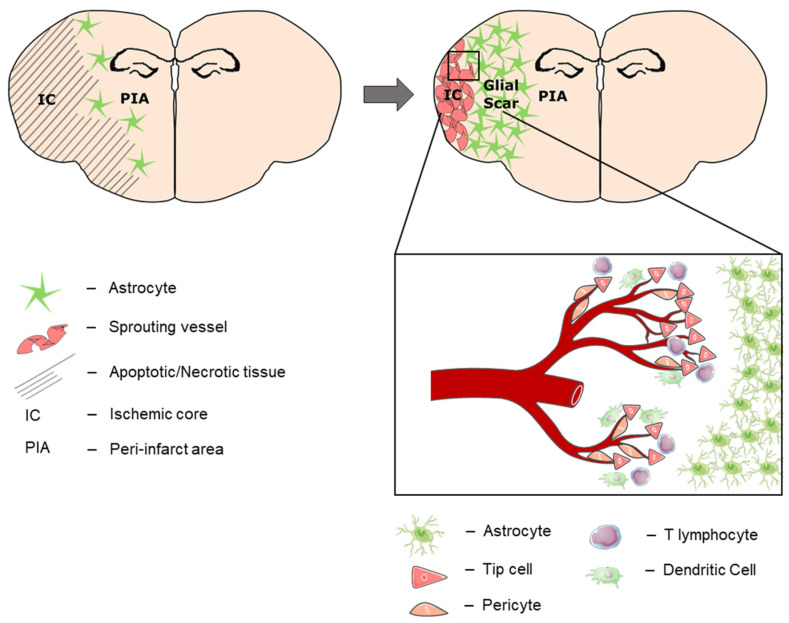
Tissue compartmentalization 28 days after stroke. During post-stroke recovery, the astroglial scar separates the affected hemisphere into the peri-infarcted area (PIA) and the ischemic core (IC). Over time, neural regeneration occurs within the PIA, which expands towards the cortical periphery as the IC shrinks. At the subpial cortex within the IC, another region of immune cell infiltration and neovascularization (IC-ANV) occurs in the late stage of post-stroke recovery.

This study explored the spatio-temporal dynamics of post-stroke angiogenesis within the ischemic core after experimental stroke. We show a clear and reproducible compartmentalization of the affected brain tissue into (a) the peri-infarct area, which is separated by (b) a broad astroglial scar from (c) the ischemic core (Figure 7). In the context of chronic remodeling, the ischemic core transforms from the cell-deprived leftovers of sudden and broad cell death to (d) an immunological active zone at the subpial periphery (“ischemic core area of neovascularization/IC-ANV”). A strong vascular reorganization occurred starting 14 days after ischemia, which exceeded mere *arteriogenesis* by sprouting *angiogenesis* within the ischemic core. Importantly, our study shows for the first time that in addition to the peri-infarct area, there is strong vasculo-immunological activity within the infarct core. We therefore postulate that the post-ischemic parenchyma is subjected to transformative processes on both sides of the astroglial scar.

In this context, the striking regenerative potential of the peri-infarct area in mice becomes apparent, which goes along with comprehensive neural repopulation of the post-ischemic hippocampus as well as the surrounding peri-infarcted parenchyma outside the astroglial scar. This correlates well with complete normalization of the animals’ clinical neurological status and indicates a remarkable capacity for adult neurogenesis in the rodent brain. Artificial selection of mildly affected animals at late post-stroke stages was ruled-out in our study due to (1) the utilization of an intensive post-stroke care protocol. At this, we achieved a mortality rate of 26%, which reflects the one of patients facing a severe stroke of the anterior cerebral circulation [37] and reduced mortality bias to the model-specific minimum. (2) By subjecting the animals to regular clinical scoring, we verified that the study population was homogenous concerning their neurological deficits. Additionally, the weight-loss curve did not show sudden “pseudo improvement” after examination days 1, 3, 7 or 14, which would retrospectively hint at selective removal of severely affected animals. (3) All mice used for this study showed cortical infarction, which was proven by cresyl violet and MAP2 staining, while at later timepoints, subcortical regions appeared largely intact. In the case of short-term or imperfect arterial occlusion, tMCAO is known to lead to subcortical infarctions, typically sparing the peripheral cortex [38,39]. Taken together, we can exclude selection bias towards smaller infarct sizes at later examination timepoints as an alternative explanation for the high density of neurons in the chronic stage of post-stroke regeneration.

Apart from this, a high degree of endothelial-cell proliferative activity developed within the ischemic cortex at late stages of post-stroke recovery. This IC-ANV was completely surrounded by the astroglial scar and was therefore clearly separated from the peri-infarct area. By immunohistochemical examination, we identified several subgroups of endothelial cells ranging from endothelial progenitor cells (EPCs) over CD31^+^/CD34^+^ tip cells to CD31^+^/CD34^−^ mature endothelial cells organized in clear tubular formations, hinting at the delayed induction of angiogenesis in this distinct zone within the IC. As the expected acute leptomeningeal *arteriogenesis*, defined as the activation of leptomeningeal collaterals, is rather dominated by the action of monocytes [40] and circulating CD31^+^/CD34^+^ stem cells would not integrate into the vessel wall as seen here [41], we postulate that this represents an additional *angiogenetic* potential of the leptomeningeal vasculature in the regenerative phase after stroke.

Differing from previous reports, we did not observe neural regeneration accompanying the *angiogenesis* in the IC-ANV, which might be due to the distance from the neural stem cell niche in the SVZ and the separation by the astroglial scar. In contrast to the peri-infarct area, the IC-ANV seems to condensate over time and might resolve completely at later timepoints in mice. Thus, the IC-ANV might represent a site of removal of necrotic tissue rather than regeneration of surviving tissue, whereas the scar serves as a separative seal between those two compartments. In this light, current attempts to modulate astrocyte polarization after stroke [42,43] should be handled carefully as a premature breakup of the compartmented structure might lead to the release of noxious agents towards the preserved brain tissue.

The assumingly leptomeningeal origin of the sprouting capillaries is interesting in that these vessels differ from the central cerebral vasculature, e.g., by the lack of an external elastic lamina [44] and by being equipped with a special blood–leptomeningeal barrier (BLMB) [45]. In contrast to the blood–brain barrier (BBB), it is not composed of a multitude of neurovascular cells but consists of a mono-cellular endothelium of regionally varying permeability [46], which seems to be a critical access point for inflammatory cells to the brain [47,48]. In this regard, it has been recently reported that before invasion of the ischemic parenchyma, immune cells aggregate within the leptomeningeal compartment adjacent to as well as inside the perivascular spaces within the subpial ischemic cortex [49,50]. Moreover, it has been shown that these invading cells access the meninges preferentially by direct vascular channels from the skull bone marrow [51]. Furthermore, there is some evidence that immune cell infiltration via the BLMB could be specifically inhibited targeting certain adhesion molecules [52].

In principle, the infiltrating immune cell populations identified by MELC analysis within the IC-ANV match previous reports in temporal dynamics and quantity [53,54]. Overall, the infiltration process within the IC-ANV seems somewhat delayed over those preceding observations; however, these studies usually refer to the peri-infarct area or to the whole ischemic hemisphere. Nevertheless, we assume the same immune–vascular crosstalk as the basis for the angiogenic processes within the IC-ANV, as has been reported for other sites of vascular remodeling [55] as neighborhood analysis revealed the close proximity of dendritic cells, T lymphocytes, and pericytes to the newly spouted vessels while glial cells such as astrocytes and microglia were more distant. An overlap of the tip-cell-associated CD31^+^/CD34^+^ co-expression with infiltrating or intravascular hematopoietic stem cells was not observed in the IC-ANV since CD31^−^/CD34^+^ were basically absent in this area.

One limitation of our study is the restricted field of view, which does not allow imaging of the complete ischemic hemisphere by which particular regional alterations might have gone unnoticed. Moreover, we can only assume the leptomeningeal origin of angiogenesis within the IC-AVN due to its strictly subpial localization. Further studies are required to characterize this vascular compartment and to identify potential therapeutic targets, e.g., for differential immunomodulatory, cytoprotective or pro-angiogenic treatment strategies. For instance, acceleration of the post-ischemic clearing processes could create a secure time window for glial-focused therapeutic strategies, which might expand the regenerative potential of the peri-infarct area towards the ischemic core. On the other hand, cytoprotective agents such as the PSD-95 modulator Nerinetide, which is currently under clinical investigation in the context of large vessel occlusion with reperfusion, might exert higher effects when delivered specifically to the site of action outside the astroglial scar.

In conclusion, we assume that in the course of chronic post-stroke regeneration, a distinct process of neovascularization originating from the leptomeningeal vasculature plays a critical role for structural and functional recovery. Further insights into the compartment-specific infiltration and action of immune cells might allow tailor-made drug design in the future to enhance either regenerative or clearance processes within the affected hemisphere.

## Supplementary Materials

The following supporting information can be downloaded at: https://www.mdpi.com/article/10.3390/cells11101659/s1, Table S1: Antibodies used for MELC analyses.
