# Supplementary material for: Behind the Wall—Compartment-Specific Neovascularisation during Post-Stroke Recovery in Mice
Source: Cells. 2022 May 17;11(10):1659. doi: 10.3390/cells11101659 (PMC9139871; doi:10.3390/cells11101659)
Supplement: Supplementary file 1 [file cells-11-01659-s001.zip › cells-1691242-supplementary.pdf]

Table S1: Antibodies used for MELC analyses.

Target	Company	Clone number	Order number
Arginase 1	Bioss		bs-8585R
CD3	Miltenyi Biotec	REAG1	130-109-838
CD4	Southern Biotech	L3T4	1540-02
CD8	BD Pharmingen	53-6.7	553030
CD11b	BioRad	M1/70.15	MCA74F
CD11c	Miltenyi Biotec	N418	130-102-799
CD29	Miltenyi Biotec	HMβ1-1	130-102-557
CD31	BD Biosciences	MEC13.3	553373
CD34	Miltenyi Biotec	REA383	130-117-775
CD45	Miltenyi Biotec	30F11.1	130-116-535
CD62E	Miltenyi Biotec	REA369	130-105-511
CD80	Biolegend	16-10A1	104706
CD86	Biolegend	GL-1	105002
Collagen IV	Novus Biologicals	(polyclonal)	NBP1-26549
F4-80	Biolegend	BM8	123107
GFAP	eBioscience	GA5	53-9892-80
Iba1	Wako	(polyclonal)	019-19741
Ki67	eBioscience	SolA15	11-5698-80
Ly6C	eBioscience	HK 1.4	17-5932-80
Ly6G	eBioscience	RB6-8C5	RM3005
MAP2	abcam	(polyclonal)	ab32454
MHC II	Miltenyi Biotec	REA813	130-112-233
NeuN	Sigma Aldrich	A60	MAB377C3
NG2	Miltenyi Biotec	REA969	130-116-141
Vimentin	Santa Cruz	V9	Sc-6260
Vinculin	Sigma Aldrich	hVIN-1	V9131
